# Perforator Selection with Computed Tomography Angiography for Unilateral Breast Reconstruction: A Clinical Multicentre Analysis

**DOI:** 10.3390/medicina60091500

**Published:** 2024-09-14

**Authors:** Ishith Seth, Bryan Lim, Robert Phan, Yi Xie, Peter Sinkjær Kenney, William E. Bukret, Jørn Bo Thomsen, Roberto Cuomo, Richard J. Ross, Sally Kiu-Huen Ng, Warren M. Rozen

**Affiliations:** 1 Department of Plastic and Reconstructive Surgery, Peninsula Health, Melbourne 3199, Australia; 2 Department of Plastic and Reconstructive Surgery, Odense University Hospital, 5000 Odense, Denmark; 3 Department of Plastic and Reconstructive Surgery, UNC School of Medicine, Chapel Hill, NC 27599, USA; 4 Plastic Surgery Unit, Department of Medicine, Surgery and Neuroscience, University of Siena, 53100 Siena, Italy; 5 Department of Plastic and Reconstructive Surgery, The Austin Health, Melbourne 3084, Australia

**Keywords:** computed tomography angiography, breast reconstruction, DIEP flap, large language models, plastic surgery, AI in medicine

## Abstract

*Background and Objectives*: Despite CTAs being critical for preoperative planning in autologous breast reconstruction, experienced plastic surgeons may have differing preferences for which side of the abdomen to use for unilateral breast reconstruction. Large language models (LLMs) have the potential to assist medical imaging interpretation. This study compares the perforator selection preferences of experienced plastic surgeons with four popular LLMs based on CTA images for breast reconstruction. *Materials and Methods*: Six experienced plastic surgeons from Australia, the US, Italy, Denmark, and Argentina reviewed ten CTA images, indicated their preferred side of the abdomen for unilateral breast reconstruction and recommended the type of autologous reconstruction. The LLMs were prompted to do the same. The average decisions were calculated, recorded in suitable tables, and compared. *Results*: The six consultants predominantly recommend the DIEP procedure (83%). This suggests experienced surgeons feel more comfortable raising DIEP than TRAM flaps, which they recommended only 3% of the time. They also favoured MS TRAM and SIEA less frequently (11% and 2%, respectively). Three LLMs—ChatGPT-4o, ChatGPT-4, and Bing CoPilot—exclusively recommended DIEP (100%), while Claude suggested DIEP 90% and MS TRAM 10%. Despite minor variations in side recommendations, consultants and AI models clearly preferred DIEP. *Conclusions*: Consultants and LLMs consistently preferred DIEP procedures, indicating strong confidence among experienced surgeons, though LLMs occasionally deviated in recommendations, highlighting limitations in their image interpretation capabilities. This emphasises the need for ongoing refinement of AI-assisted decision support systems to ensure they align more closely with expert clinical judgment and enhance their reliability in clinical practice.

## 1. Introduction

Computed tomography angiograms (CTAs) play a pivotal role in the preoperative planning of DIEP (Deep Inferior Epigastric Perforator) and TRAM (Transverse Rectus Abdominis Myocutaneous) flaps used in breast reconstruction surgeries [1,2]. These detailed imaging studies provide essential information on vascular anatomy, enabling surgeons to identify the most suitable perforator vessels for flap survival and minimising complications [1,2]. However, despite reviewing the same CTA images, experienced plastic surgeons may have varying preferences regarding which side of the abdomen to use for unilateral breast reconstruction, influenced by their training, experience, and individual interpretation of the images [3].

Large language models (LLMs) like ChatGPT-4 are increasingly gaining traction in the medical field because of their ability to assist with a range of tasks, including the interpretation of medical imaging such as CTAs [4,5]. These models offer consistent, data-driven insights and can be valuable decision-support tools in clinical practice. By leveraging advanced AI capabilities, LLMs have the potential to enhance diagnostic accuracy, streamline workflows, and provide supplementary opinions that may help in complex decision-making processes [6,7,8].

This multicentric study aims to compare the perforator selection preferences of experienced plastic surgeons with those of ChatGPT-4 based on CTA images for breast reconstruction. Understanding these differences will highlight the current capabilities and limitations of AI in replicating expert clinical judgment. By identifying areas where AI aligns with or diverges from human expertise, the findings can inform future AI developments, ultimately improving patient outcomes and aiding surgeons in making more informed decisions.

## 2. Materials and Methods

Six experienced specialist plastic surgeons from Australia, USA, Italy, Denmark, and Argentina reviewed ten two-dimensional CTA images (Supplementary File S1) from ten de-identified female patients, with an average age of 58 years. While the majority of patients were Caucasian, the cohort included a range of ethnicities, including African, Australian, and European. They indicated their preferred side of the abdomen for unilateral breast reconstruction and the type of breast reconstruction they would recommend (Table 1). This involved a detailed analysis of the vascular anatomy provided by the CTA images to decide which operation is most suitable. No institutional ethics approval was required for this study. However, authorisation was obtained from the radiological company for the use of de-identified CTA images from their patient database.

The same images were analysed by various LLMs, including ChatGPT-4 (OpenAI, San Francisco, CA, USA), ChatGPT-4o (OpenAI, San Francisco, CA, USA), Co-Pilot (Microsoft, Redmond, WA, USA), and Claude (Anthropic, San Francisco, CA, USA). Each model was prompted with the same prompt, “Analyse these images to determine the most suitable side of the abdomen for breast reconstruction and recommend the type of autologous breast reconstruction you would perform for this patient”, to determine the most suitable side of the abdomen for reconstruction and recommend the type of autologous breast reconstruction (Table 2). This process required a detailed analysis of the vascular anatomy provided by the static CTA images to determine which side offers the best perforators for flap survival. Due to the inability to upload complete CTA videos to LLMs, static images were utilised for this study. The use of static images aimed to replicate the decision-making process as closely as possible given the technological constraints. LLMs analyse static images indirectly by processing detailed textual descriptions of the image content. Unlike image-specific AI models, LLMs do not directly interpret visual data. Instead, they rely on text-based inputs that describe image features, such as anatomical landmarks or patterns. These descriptions are processed to generate insights or recommendations. The analysis is limited to the information provided in the text, and LLMs lack the capability to directly assess visual details, such as subtle variations in texture or spatial relationships. Therefore, their utility in image analysis depends on the accuracy and completeness of the textual descriptions they receive. Therefore, we hypothesis that LLMs analyse static CTA 2-D images by focusing on textual descriptions of key features such as the vascular anatomy, including the location and quality of perforator vessels and relevant anatomical landmarks. These descriptions guide the LLMs in recommending the most suitable side of the abdomen for breast reconstruction and the appropriate surgical technique. Furthermore, they may prioritise factors like vessel viability and anatomical symmetry to determine their recommendations, with a strong preference for the DIEP flap based on the provided textual data.

**Figure 1:**
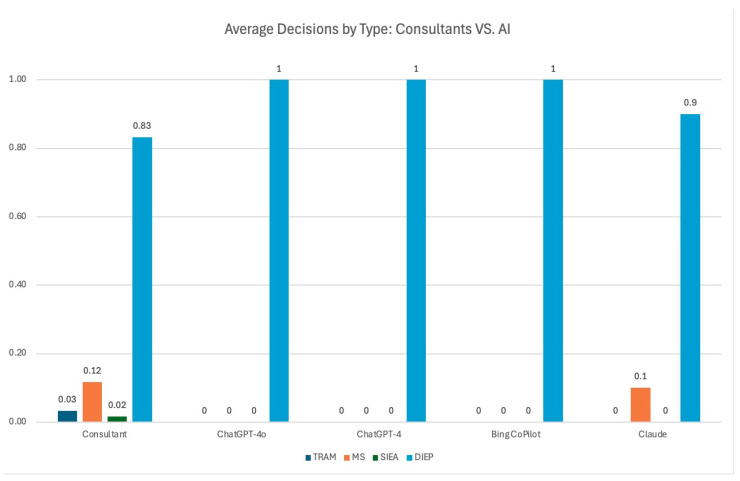
Average decisions between experienced plastic surgeons and artificial intelligence on unilateral breast reconstruction based on CTA perforator.

The total number of responses per participant group (consultants, ChatGPT-4o, ChatGPT-4, Co-Pilot, and Claude) was recorded. The average decision for each recommendation was calculated by dividing the total number of specific recommendations by the total number of participants, expressed as numerical values (Table 3) and graphically represented (Figure 1). For instance, if five consultants made 42 DIEP flap decisions out of 50 scenarios, the average decision rate for consultants would be 42 divided by 50, resulting in an average decision rate of 0.84.

## 3. Results

Six consultants responded, originating from diverse locations, including Italy, USA, Argentina, Denmark, and Australia. The predominant recommendation was the DIEP procedure, with “Right DIEP” being most frequently advised in scenarios 1, 3, 4, and 7, ranging from 67% to 100% of the recommendations. Conversely, “Left DIEP” was the primary choice in scenarios 2, 5, 6, 9, and 10, ranging from 67% to 100%. Consultants predominantly favoured the DIEP procedure, with 83.33% recommending it, followed by fewer instances of recommendations for MS TRAM (11.67%), SIEA (1.67%), or TRAM (3.33%), indicating a secondary preference for these procedures. The variations highlight the consultants’ inclination towards either right or left DIEP procedures, with occasional consideration for alternative methods based on specific case details.

ChatGPT-4o exclusively recommended the DIEP procedure, with 100% of its recommendations being for DIEP. ChatGPT-4 also exclusively recommended DIEP, showing a consistent preference for this approach. Bing CoPilot, like ChatGPT-4o and ChatGPT-4, recommended DIEP procedures exclusively, emphasising its preference for this technique. Claude’s recommendations included 90% for DIEP and 10% for MS TRAM, making it the only model to suggest an alternative procedure. Across all LLMs, there appeared to be a unanimous preference for the DIEP technique, ranging from 90% to 100% of the recommendations. Claude was the only exception by occasionally considering an alternative method.

These images were flipped horizontally and uploaded to the LLMs to assess consistency in their recommendations. Interestingly, ChatGPT-4o flipped its preference side, providing recommendations opposite to its initial ones yet still exclusively recommending the DIEP procedure in 100% of cases. This suggests that ChatGPT-4o might interpret the images more effectively by consistently recognising anatomical landmarks and vascular patterns, regardless of image orientation. Such performance indicates potential superiority in image analysis, highlighting the need for further research into the algorithms underpinning ChatGPT-4o’s interpretive capabilities.

## 4. Discussion

This study reveals that consultants consistently preferred the DIEP procedure for unilateral breast reconstruction. This reflects experienced surgeons’ confidence and comfort in raising DIEP flaps over other techniques like TRAM flaps. The analysis of the same static CTA images by LLMs, including ChatGPT-4, ChatGPT-4o, Co-Pilot, and Claude, showed a unanimous preference for the DIEP procedure as well. However, when the images were flipped horizontally, ChatGPT-4o notably provided recommendations opposite to its initial ones, suggesting it may interpret images more effectively by consistently recognising anatomical landmarks and vascular patterns regardless of orientation. This performance highlights ChatGPT-4o’s potential superiority in image analysis and underscores the necessity for further research to refine and validate AI-assisted decision support systems to align with expert clinical judgment and enhance their reliability in clinical practice.

The recommendations provided by each LLM revealed nuanced preferences in breast reconstruction techniques, which ideally should have aligned with those of experienced consultants. As LLMs were trained on data generated by humans, their recommendations ideally reflected the collective expertise of consultants. For instance, in scenario one, while consultants unanimously recommended “Right DIEP”, ChatGPT-4o deviated by suggesting “Left DIEP”, indicating a discrepancy between the model’s recommendation and human expertise.

Across all scenarios, a clear preference for the DIEP procedure emerged among consultants, a trend mirrored by the LLMs. This alignment is evident in scenario 3, where consultants unanimously recommended “Right DIEP”, consistent with ChatGPT-4o’s suggestion. However, there were variations in recommendations between left and right DIEP across all groups. For example, in scenario five, while consultants leaned towards “Left DIEP”, ChatGPT-4 recommended “Right DIEP”, highlighting interpretation differences between humans and LLMs. Bing CoPilot and Claude occasionally recommended alternative procedures like “Right MS TRAM” in scenario eight, diverging from consultants’ preference for DIEP.

Comparing the recommendations of consultants with those of the LLMs revealed interesting insights. While consultants predominantly recommended right DIEP, the distribution between left and right DIEP among LLMs varied. This discrepancy is illustrated in scenario 7, where most consultants recommended “Right DIEP”, but Bing CoPilot suggested “Left DIEP”. Similarly, in scenario 8, while consultants leaned towards “Right DIEP”, ChatGPT-4 suggested “Left DIEP”, indicating differences in prioritisation and interpretation. Notably, the MS TRAM procedure was rarely recommended across all groups, indicating a limited consideration of this technique. This alignment is evident in scenario nine, where none of the consultants recommended MS TRAM, which is consistent with the overall trend observed with LLMs. However, it also raised questions about the factors influencing this preference. The predominance of DIEP flap recommendations by LLMs likely stems from biases in their training data, which may emphasise this technique due to its widespread use and positive outcomes reported in the recent surgical literature. These models are designed to identify and replicate frequent patterns within their datasets, and the DIEP flap’s advantages in preserving abdominal muscles and achieving natural aesthetic results likely influenced the AI’s decisions. Additionally, lacking nuanced clinical judgment, LLMs may default to the most commonly recommended procedure in their dataset. This underscores the importance of further research to explore the efficacy and suitability of different surgical techniques in breast reconstruction.

The findings highlight significant limitations in the current capabilities of LLMs, particularly in tasks involving image interpretation [9,10]. Unlike human observers, LLMs lack the visual acuity and interpretive skills to accurately assess intricate anatomical details in medical images such as CT angiograms [11]. This inherent limitation poses challenges in replicating the nuanced clinical judgment and contextual understanding that experienced plastic surgeons possess. Consultants are adept at interpreting medical images within the broader clinical picture, integrating patient history and clinical findings for accurate diagnosis and treatment planning. They utilise a combination of clinical judgment, intuition, and patient interaction to make decisions, including the ability to interpret patient preferences and psychological readiness for procedures. AI, such as ChatGPT-4, although proficient in pattern recognition, may miss these critical contextual cues, leading to disparities in recommendations compared to seasoned practitioners. These insights emphasise the need for ongoing refinement and validation of AI-assisted decision support systems in clinical practice to ensure optimal patient outcomes and aid surgeons in making informed decisions [12,13].

During the prompting phase, Bing CoPilot and Claude often refrained from providing medical recommendations, introducing significant implications for clinical practice and user experience. Firstly, the reluctance or inability of LLMs to offer medical recommendations suggests potential limitations in their understanding or confidence in providing clinical guidance. This may stem from constraints in their training data or inherent biases in their algorithms, leading to a reluctance to offer definitive advice in medical contexts [11,14]. Consequently, clinicians relying on such models may encounter obstacles in obtaining timely and accurate decision support, potentially impacting patient care and clinical outcomes. Conversely, the ChatGPT models demonstrated a faster response when providing recommendations. This efficiency in delivering insights could streamline decision-making processes in clinical practice, enabling clinicians to access relevant information more rapidly. This accelerated workflow could be particularly beneficial in time-sensitive situations or when clinicians require quick access to supplementary opinions to inform their decisions [15,16]. Consultants, on the other hand, can adapt their recommendations based on evolving knowledge and new clinical guidelines. This allows them to incorporate the latest research, advancements in surgical techniques, and changes in best practices, ensuring their decisions align with current standards of care. This adaptability ensures optimal patient outcomes and high-quality care.

The differences in response times and willingness to provide medical recommendations among AI models could also challenge user experience and trust [17,18]. Clinicians would prefer models that consistently offer prompt and reliable recommendations, possibly leading to frustration or scepticism towards models that exhibit hesitancy or delays in providing guidance. Moreover, inconsistencies in the performance of AI models may undermine users’ confidence in their reliability and suitability for clinical decision support, potentially hindering their adoption and integration into routine practice [18].

Beyond the visible vascular anatomy assessed through CTA, several critical factors are considered when selecting the side for breast reconstruction. These include the quality and thickness of the abdominal tissue, the presence of any previous scars or surgical interventions that might impact flap viability, and the relative distribution of perforator vessels on each side. Surgeons also evaluate patient-specific factors such as body habitus, overall health, and any comorbid conditions that could affect healing and outcomes. Additionally, the potential for donor site morbidity, the need for symmetry with the contralateral breast, and the patient’s aesthetic preferences are integral to the decision-making process, ensuring a balanced approach that optimises both functional and cosmetic results.

To address the limitations observed in LLMs like Bing CoPilot and Claude, several improvements are necessary to enhance their effectiveness and usability in clinical practice. Firstly, comprehensive training data encompassing diverse medical scenarios and contexts are essential to ensure that LLMs have a robust understanding of clinical decision-making [19,20]. Secondly, algorithms must be refined to minimise biases and enhance the models’ ability to provide accurate and reliable medical recommendations [21,22,23,24,25]. Transparent communication about the capabilities and limitations of AI systems is crucial to managing user expectations and fostering trust among clinicians. Lastly, ongoing evaluation and validation of AI models in real-world clinical settings can help identify areas for improvement and ensure that these systems continue to evolve and adapt to the dynamic healthcare landscape [26,27]. Additionally, in this study, the personal experiences and regional practices of the participating surgeons, all with over 10 years of experience, likely played a significant role in their decision-making. Surgeons develop preferences based on their clinical exposure, regional training, and the common practices within their geographic area. These factors influence their interpretation of CTA images and the selection of surgical techniques. For example, familiarity with specific flap procedures or regional trends in breast reconstruction may lead to a preference for certain approaches. Thus, their decisions are shaped by a combination of individual expertise and the prevailing surgical practices in their respective regions.

Lastly, to address potential biases in the training data influencing LLMs’ recommendations, it is crucial to ensure the dataset’s diversity, encompassing various demographic and geographic profiles. Bias mitigation techniques, such as balanced sampling and algorithmic adjustments, are employed to reduce over-representation of specific groups. Continuous monitoring and validation against real-world clinical decisions are necessary to detect and correct biases, ensuring that the LLMs’ outputs align closely with expert clinical judgment and diverse patient populations.

## 5. Conclusions

This study highlights the preferences of experienced plastic surgeons and LLMs in selecting perforator vessels for breast reconstruction. Both consultants and LLMs predominantly favoured DIEP procedures, reflecting their confidence in this technique. LLMs, including ChatGPT-4, ChatGPT-4o, Co-Pilot, and Claude, also showed a strong preference for DIEP, with ChatGPT-4o notably flipping its recommendations when images were horizontally flipped, indicating a nuanced understanding of anatomical landmarks. These findings suggest that while LLMs can effectively replicate human preferences, there are still limitations, particularly in image orientation consistency. Addressing these limitations requires comprehensive training data, algorithm refinement, and transparent communication to enhance the effectiveness of AI-assisted decision support systems in clinical practice.

## Figures and Tables

**Table 1:** Experienced plastic and reconstructive consultants’ decisions on unilateral breast reconstruction based on CTA perforator.

Scenario	Consultant 1	Consultant 2	Consultant 3	Consultant 4	Consultant 5	Consultant 6
1	Right DIEP	Right DIEP	Right DIEP	Right DIEP	Right DIEP	Right DIEP
2	Left DIEP	Right DIEP	Right MS TRAM	Right DIEP	Left MS TRAM	Left DIEP
3	Right DIEP	Right DIEP	Right DIEP	Right DIEP	Right DIEP	Right DIEP
4	Right DIEP	Right DIEP	Right DIEP	Right MS TRAM	Right MS TRAM	Right DIEP
5	Left DIEP	Right DIEP	Right MS TRAM	Left DIEP	Left DIEP	Left DIEP
6	Left DIEP	Right DIEP	Left DIEP	Left DIEP	Right DIEP	Left DIEP
7	Right DIEP	Left DIEP	Left DIEP	Right DIEP	Right DIEP	Right DIEP
8	Left MS TRAM	Right DIEP	Left TRAM	Left SIEA	Left TRAM	Left MS TRAM
9	Left DIEP	Left DIEP	Left DIEP	Left DIEP	Left DIEP	Left DIEP
10	Left DIEP	Left DIEP	Left DIEP	Left DIEP	Left DIEP	Left DIEP

**Table 2:** Large language models’ decisions on unilateral breast reconstruction based on CTA perforator.

Scenario	ChatGPT4o	ChatGPT4	Bing CoPilot	Claude
1	Left DIEP	Right DIEP	Right DIEP	Right DIEP
2	Left DIEP	Right DIEP	Right DIEP	Left DIEP
3	Right DIEP	Right DIEP	Left DIEP	Right DIEP
4	Left DIEP	Right DIEP	Left DIEP	Left DIEP
5	Right DIEP	Left DIEP	Left DIEP	Right DIEP
6	Right DIEP	Left DIEP	Right DIEP	Right DIEP
7	Left DIEP	Left DIEP	Right DIEP	Left DIEP
8	Left DIEP	Right DIEP	Right DIEP	Right MS TRAM
9	Right DIEP	Right DIEP	Right DIEP	Left DIEP
10	Right DIEP	Right DIEP	Right DIEP	Right DIEP

**Table 3:** Average decisions by type: consultants versus artificial intelligence.

	TRAM	MS	SIEA	DIEP
Consultant	0.03	0.11	0.02	0.83
ChatGPT-4o	0	0	0	1
ChatGPT-4	0	0	0	1
Bing CoPilot	0	0	0	1
Claude	0.1	0	0	0.9

## Data Availability

Data can be available upon reasonable request to the corresponding author.

## Supplementary Materials

The following supporting information can be downloaded at https://www.mdpi.com/article/10.3390/medicina60091500/s1, Supplementary File S1—CTA images of all ten patients.
