# Supplementary material for: Perforator Selection with Computed Tomography Angiography for Unilateral Breast Reconstruction: A Clinical Multicentre Analysis
Source: Medicina (Kaunas). 2024 Sep 14;60(9):1500. doi: 10.3390/medicina60091500 (PMC11433981; doi:10.3390/medicina60091500)

CTA of patient 1

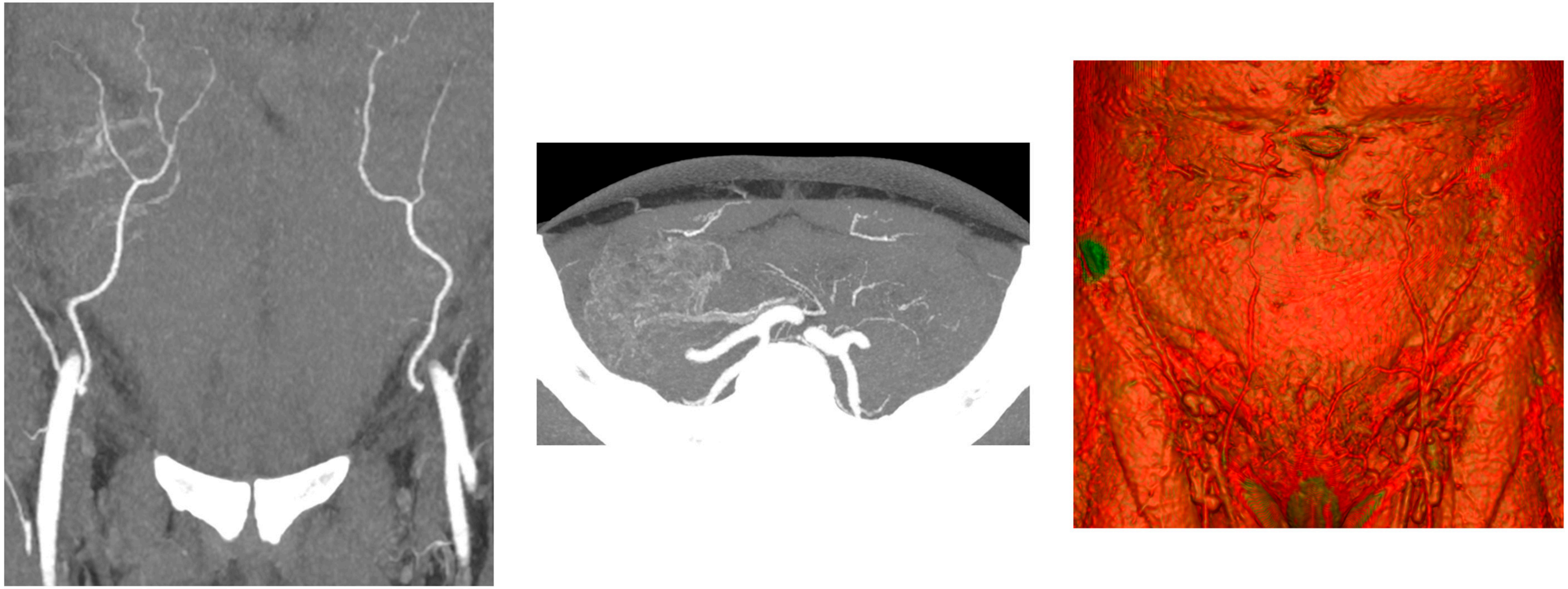

CTA of patient 2

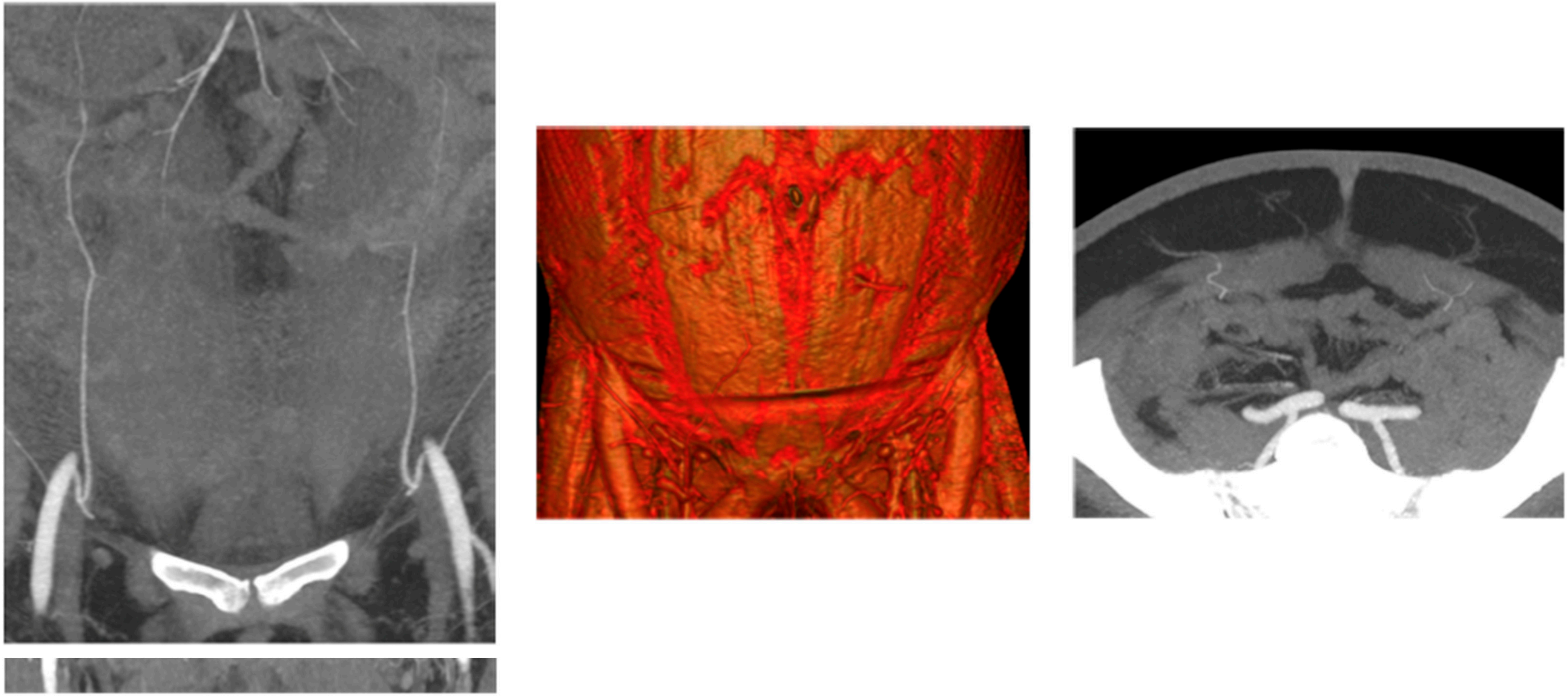

CTA of patient 3

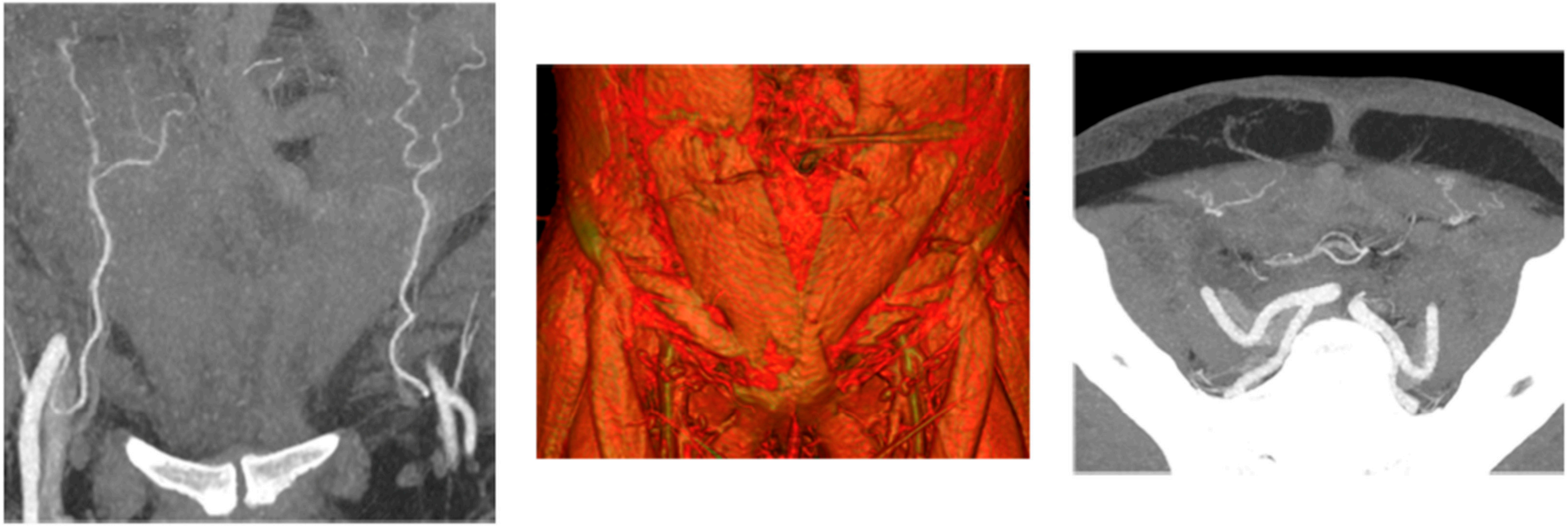

CTA of patient 4

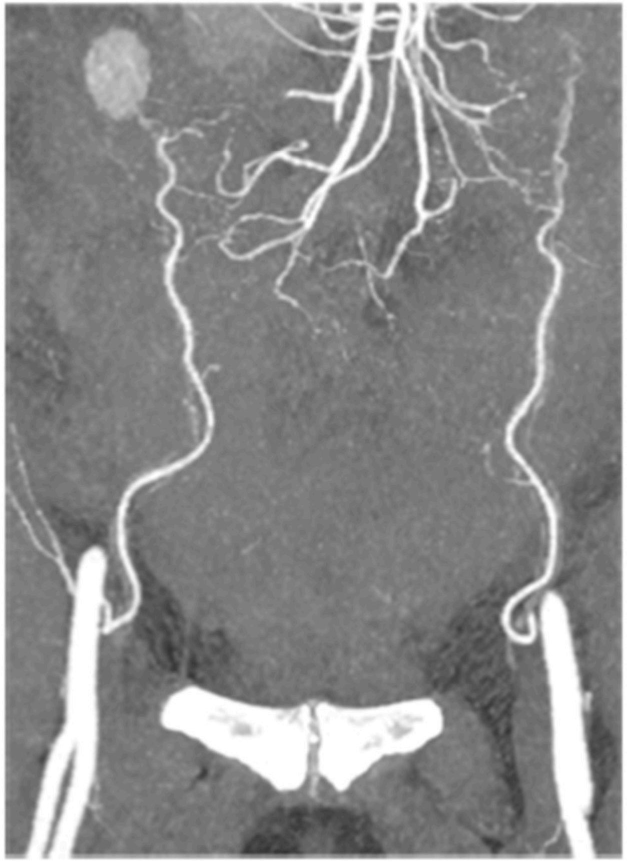

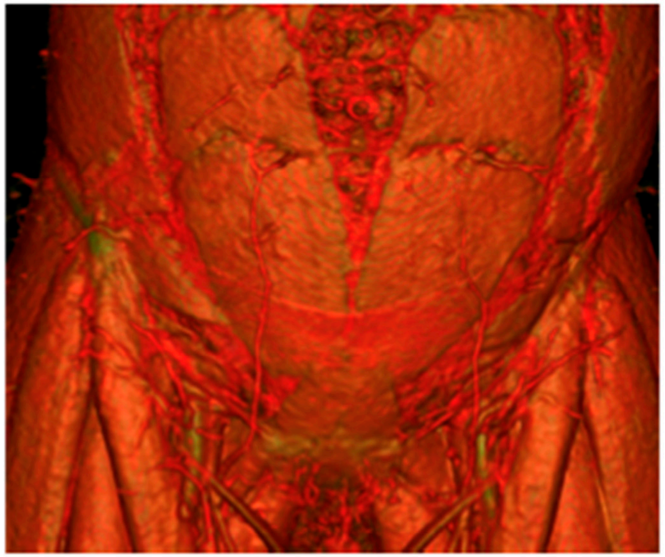

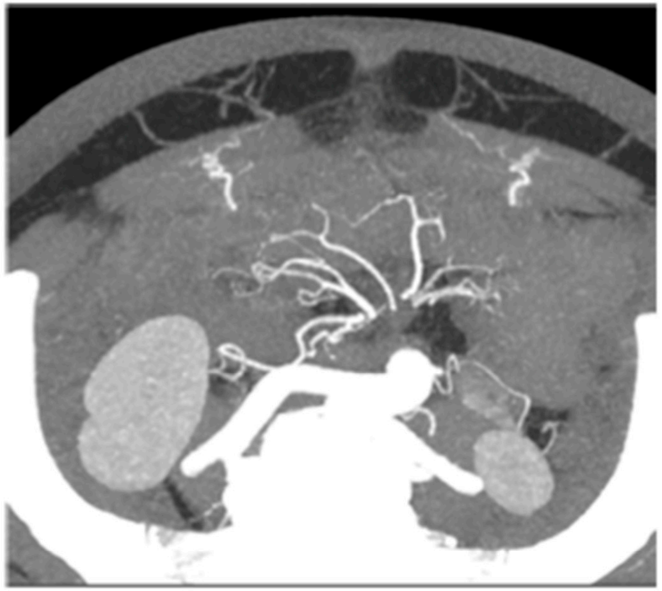

CTA of patient 5

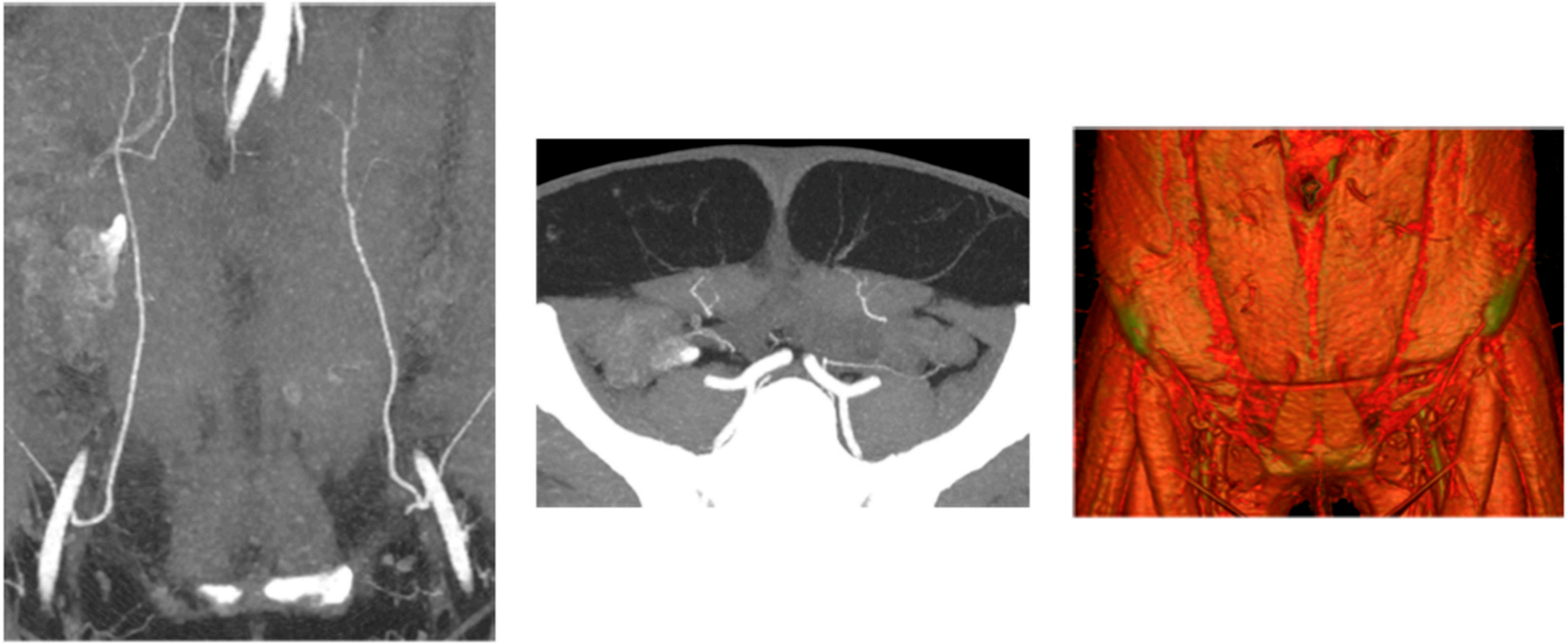

CTA of patient 6

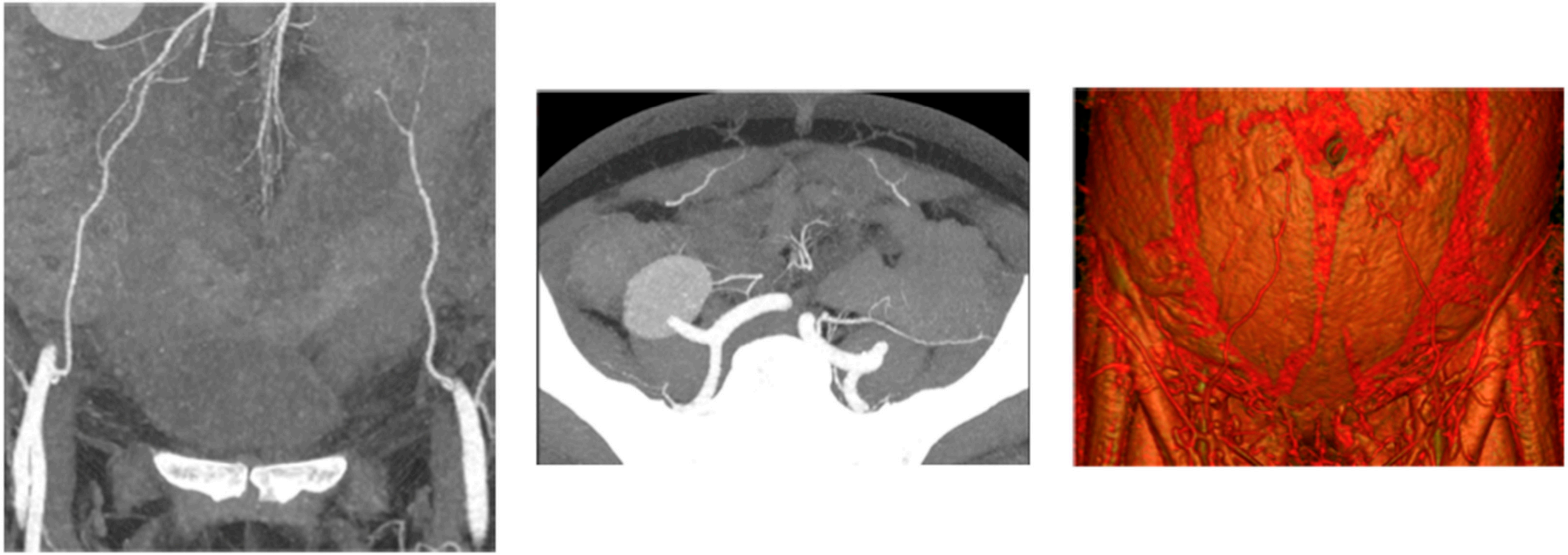

CTA of patient 7

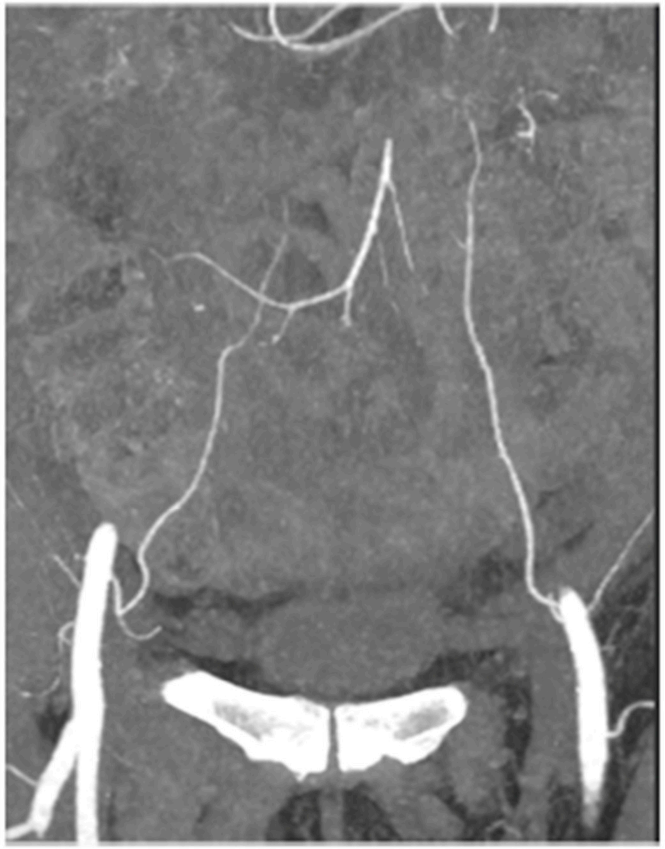

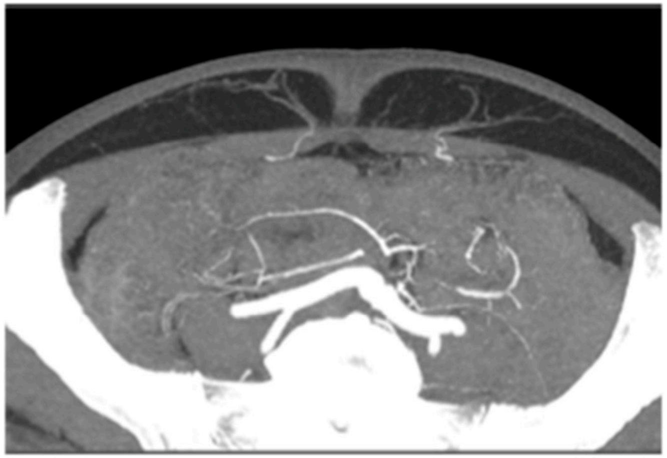

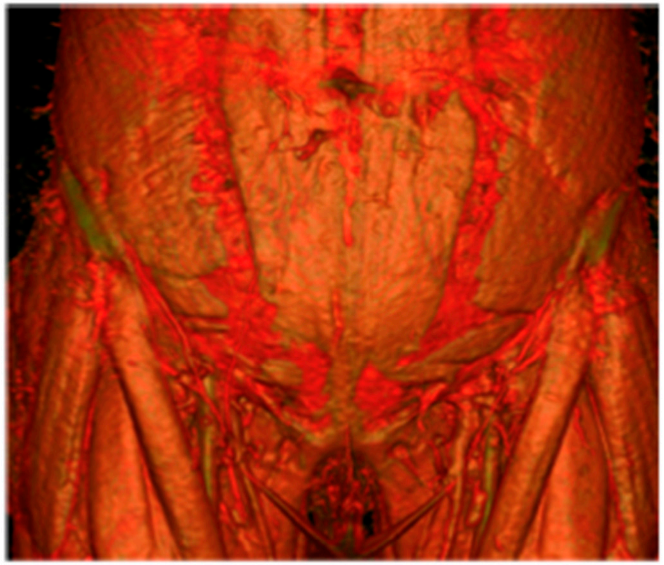

CTA of patient 8

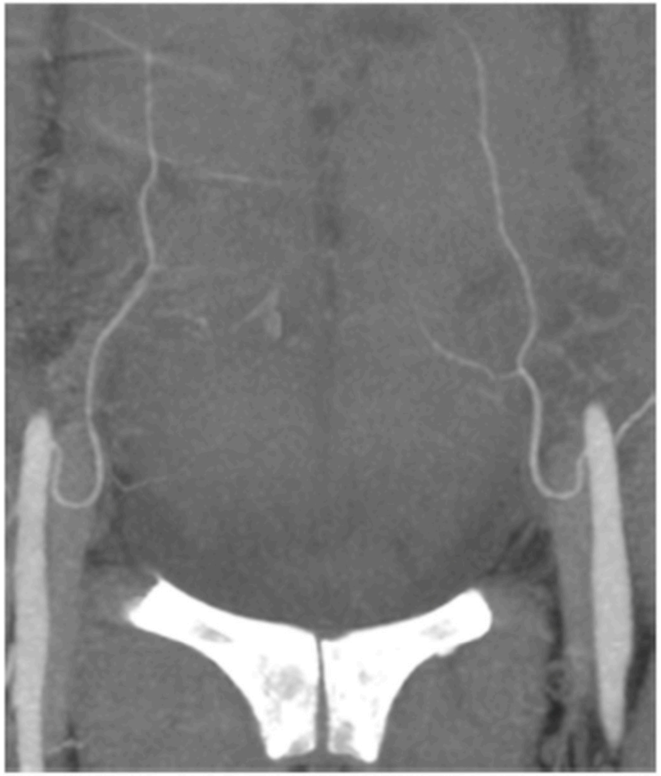

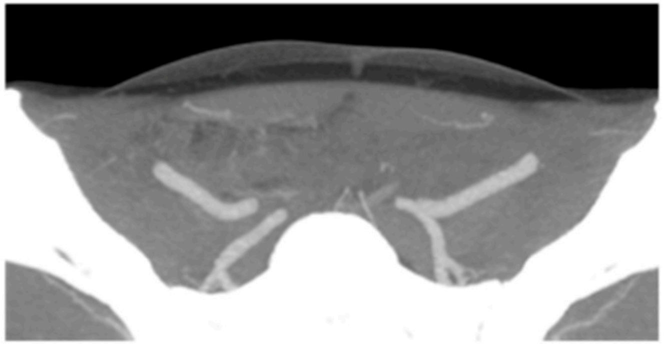

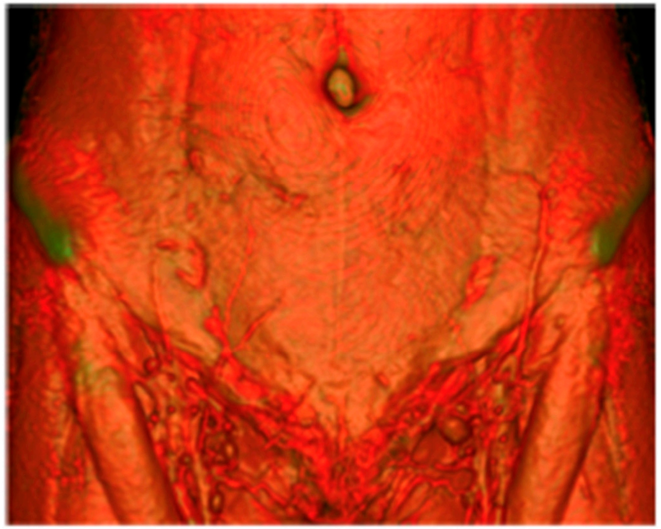

CTA of patient 9

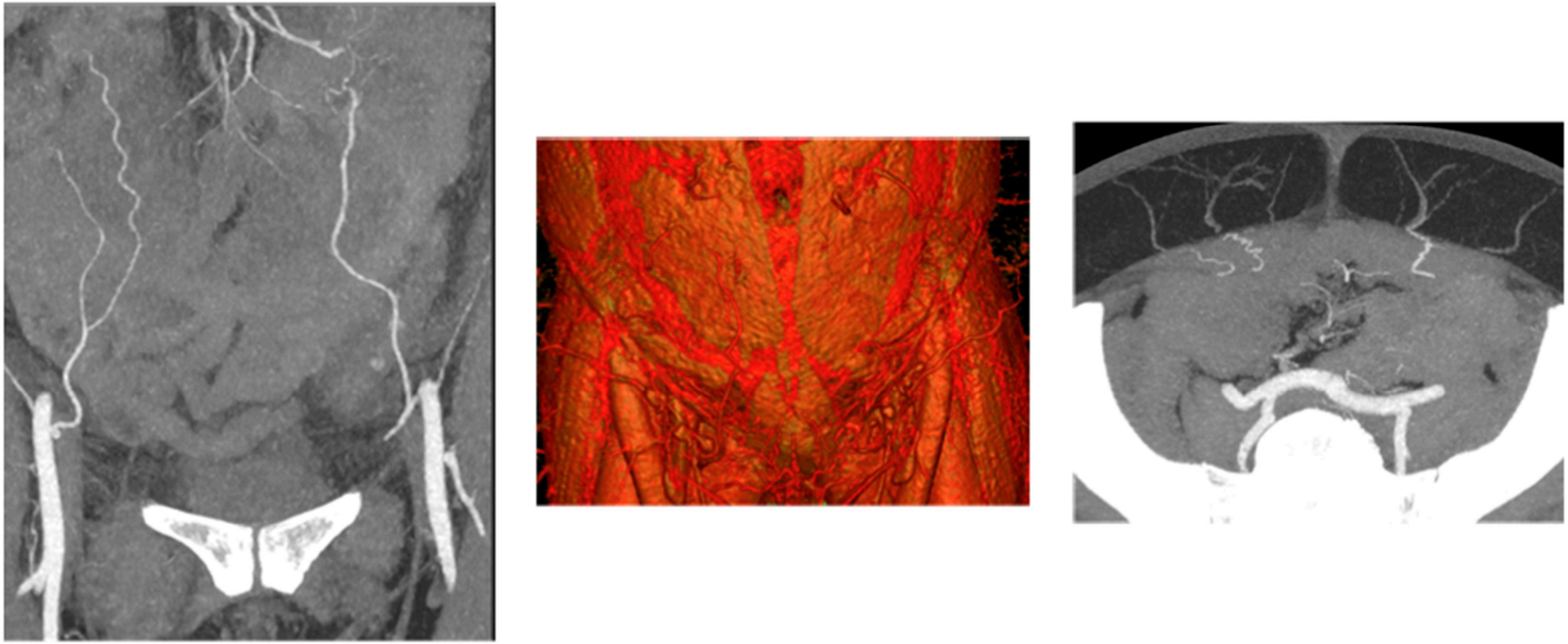

CTA of patient 10

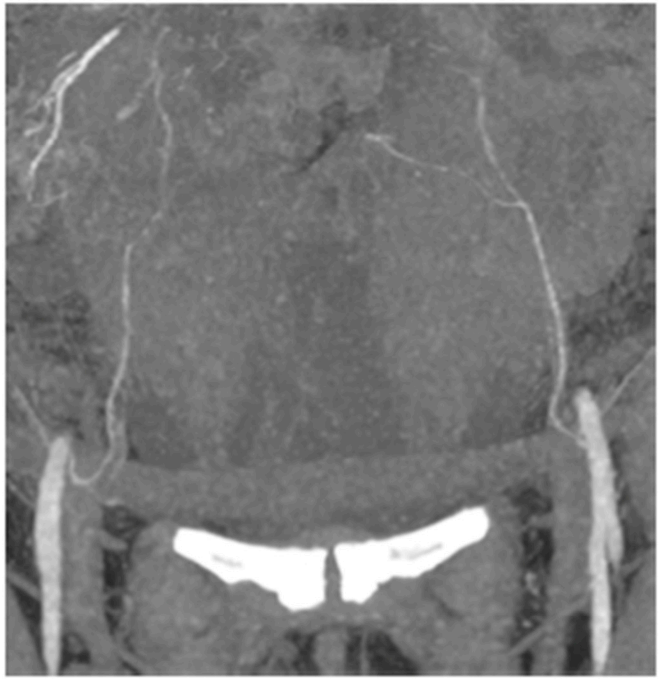

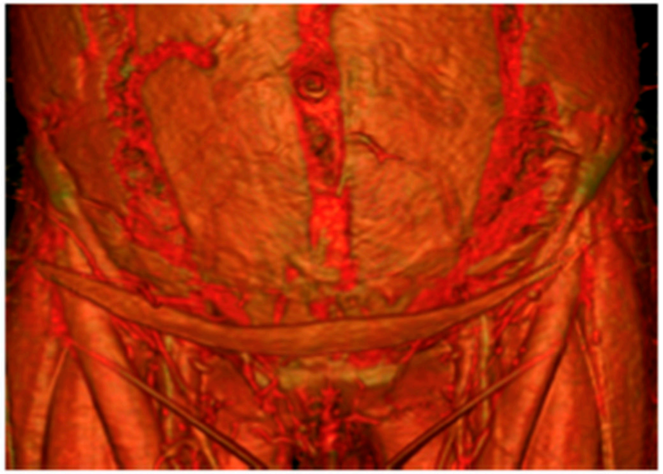

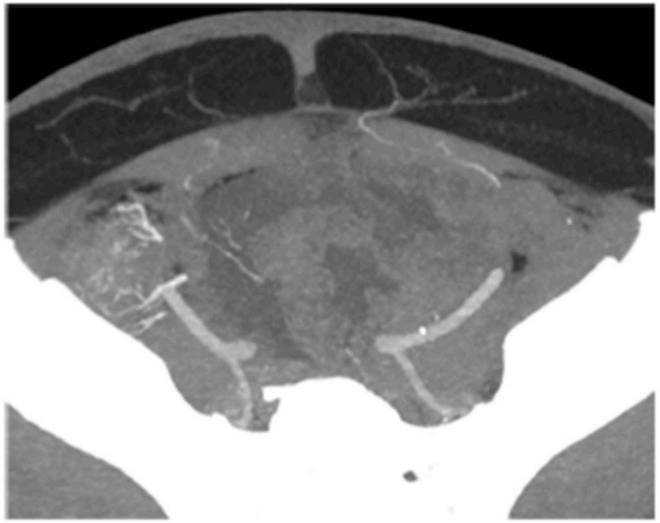

Supplement: Supplementary file 1 [file medicina-60-01500-s001.zip › medicina-3167085-supplementary.pdf]
